# Variation rs9929218 and risk of the colorectal Cancer and adenomas: A meta-analysis

**DOI:** 10.1186/s12885-021-07871-z

**Published:** 2021-02-24

**Authors:** Huiyan Wang, Dongying Gu, Miao Yu, Yanjun Hu, Zhe Chen, Xinying Huo, Tao Yu, Jinfei Chen, Yang Zheng

**Affiliations:** 1 Liaoning Cancer Hospital of China Medical University, Liaoning Cancer Hospital & Institute, No.44 Xiaoheyan Road, Dadong District, Shenyang, Liaoning Province 110042 People’s Republic of China; 2 Department of Oncology, The Affifiliated Nanjing First Hospital, Nanjing Medical University, 68 Changle Road, Nanjing, People’s Republic of China

**Keywords:** Colorectal cancer, Adenomas, SNP, rs9929218, Meta-analysis

## Abstract

**Backgrounds:**

Genome-wide association studies (GWAS) have identified multiple common CRC-related (colorectal cancer) SNPs (single nucleotide polymorphisms) including the Cadherin 1(CDH1) rs9929218 may act by increasing the risk of colorectal cancer, colorectal adenoma, or both. These studies, however, reported inconsistent associations.

**Methods:**

To derive a more accurate approximation of the connection, we carried out a meta-analysis of 12 published pieces of research including 11,590 controls and 8192 cases. We used odds ratios (ORs) and 95% confidence intervals (CIs) to evaluate the associations’ strength.

**Results:**

Meta-analysis implied considerable association between CRC and rs9929218 (OR = 1.21, 95%CI 1.04–1.42 for GG versus AA; OR = 1.22, 95%CI 1.05–1.42 for GG/AG versus AA). In the subgroup analyses, significantly increased risks were found among Europeans.

**Conclusions:**

In summary, our meta-analysis studies in different populations confirmed that SNP rs9929218 is significantly associated with CRC risk and that this variant may have a greater impact on Europeans.

## Background

More than one million people worldwide are affected by colorectal cancer (CRC) every year [1]. It is currently the third most frequent malignancy and the fourth commonest cause of cancer-related mortality in the world [2], and accounts for approximately 630,000 death from CRC annually. Previous genetic epidemiological studies provided evidence that CRC is a complex disease influenced by environmental and genetic factors and their interactions [3]. The majority of CRC are developed from colorectal adenomas (CRA) [4]. It is well-recognized that the high genetic risk of CRC is due in large part to the susceptibility to adenomas [5]. Single nucleotide polymorphism associated with CRC may increase the risk of colorectal cancer, colorectal adenomas, or both.

Genome-wide Association Studies (GWAS), effectively apply to multiple common single nucleotide polymorphisms (SNPs), and these SNPs have been illustrated to correlate with the individual susceptibility to CRC [1, 6–8]. Various kinds of genetic loci associated with increased or decreased risk of colorectal cancer (CRC) on 8q23.3, 8q24.21, 9p24, 10p14, 11q23.1, 14q22.2, 15q13.3, 16q22.1, 18q21.1, 19q13.1, and 20p12.3 have been identified by GWAS, illustrating, the CRC as a complex genetic disease [1, 6, 8–12].

Among these SNPs, rs9929218 (16p22.1), located in the intron region of the gene cadherin 1 (CDH1), was identified to be associated with CRC risk [13]. In 2016, Han et al. conducted a meta-analysis of rs9929218 including 16 studies (*n* = 131,768) which emphasizes a significant association between rs9929218 polymorphism and CRC susceptibility [13]. Nonetheless, its limitation is the absence of raw genotype data that reference dominant and recessive models.

The rs9929218 has been identified as an aroused general interest for CRC susceptibility by recent genome-wide association studies and this polymorphism has shown that the G allele is associated with an increased risk of colorectal cancer. However, some of the literature has produced contrary results [14, 15].

We, therefore, performed a meta-analysis of the published studies to clarify this antilogy and constitute a comprehensive map of the relationship between 16q22.1 (rs9929218) polymorphism and the CRC susceptibility.

## Methods

Our meta-analysis is reported followed the guideline of the PRISMA (Preferred Reporting Items for Systematic Review and Meta-Analysis) statement [16]. There are no ethical issues involved in our study because our data were based on published studies, and no ethical issues were involved in the selection, extraction and analysis of the data.

### Identification and eligibility of relevant studies

Embase, PubMed, and ScienceDirect database were hunted invoking the semesters: ‘rs9929218’, ‘Single Nucleotide Polymorphism ‘,'colorectal cancer ‘and ‘colorectal adenoma ‘to gather eligible articles. The combinations of following keywords were used: ‘Neoplasms, Colorectal’ or ‘Colorectal Neoplasm’ or ‘Neoplasm, Colorectal’ or ‘Colorectal Tumors’ or ‘Colorectal Tumor’ or ‘Tumor, Colorectal’ or ‘Tumors, Colorectal’ or ‘Colorectal Carcinoma’ or ‘Carcinoma, Colorectal’ or ‘Carcinomas, Colorectal’ or ‘Colorectal Carcinomas’ or ‘Colorectal Cancer’ or ‘Cancer, Colorectal’ or ‘Cancers, Colorectal’ or ‘Colorectal Cancers’; ‘Nucleotide Polymorphism, Single’ or ‘Nucleotide Polymorphisms, Single’ or ‘Polymorphisms, Single Nucleotide’ or ‘Single Nucleotide Polymorphisms’ or ‘SNPs’ or ‘Single Nucleotide Polymorphism’; ‘rs9929218’.

We searched literature published before February 2020 and the language was restricted to English. This search was implemented by two students independently to determine potential publications meeting our inclusion criteria. If there is a clash, two students consult with the third reviewer and achieve a consensus [3, 17]. Studies in our meta-analysis ought to meet the following inclusion standards: (1) assess the association between colorectal cancer risk and the rs9929218 polymorphism; (2) the studies were designed as case-control studies; (3) include the detailed frequency of each genotype.

### Data extraction

Two researchers appraised and extracted all data from all qualified publications independently, as reported by the inclusion standards that were listed above. The following data was gathered from each study: the name of the first author, the year of publication, the country of study, the specific number of genotypes in each control and case group; and the source of the control group (population- or hospital-based controls). Varying ethnic ancestries were classified as European, Asians, or Other including more than one ethnicity’s subjects [13, 17]. There is no duplicate sample was included in these 12 studies.

### Statistical analysis

The strength of the association between the CRC risk and rs9929218 polymorphism was measured through odds ratios (ORs) with 95% confidence intervals (CIs). The statistical significance of the pooled OR was determined using the Z-test [17]. First of all, we measured four genetic models: the homozygous model (GG vs. AA), the heterozygous model (GA vs. AA), the dominant model (GG + GA vs. AA), and the recessive model (GG vs. GA + AA) respectively (A: wild allele; G: mutated allele). Stratified analysis based on race, disease type, and source of control.

In return for the probability of heterogeneity throughout the researches, a statistical test for heterogeneity was performed based on the chi-square-based Q test [17, 18]. *P* < 0.05 was considered significant for the heterogeneity. We used two kinds of meta-analysis models including the Mantel-Haenszel (M-H) fixed-effect model and the DerSimonian-Laird (D-L) random-effect model to compute the pooled OR. With the proviso that no significant heterogeneity (I^2^ is 50% or less) learnings involved in, the pooled OR is calculated by the fixed-effect model; otherwise (I^2^ more than 75%) the OR is calculated by the random-effect model [17]. We did a sensitivity analysis to evaluate the stability of the results, one single study in the meta-analysis was excluded each time to report the influence of the respective data set to the pooled OR. We offer a potential publication bias with a funnel plot of diagnosis. All analyses were performed using STATA software (Stata Corporation, College Station, TX; version 12.0).

## Results

### Characteristics of studies

**Fig. 1:**
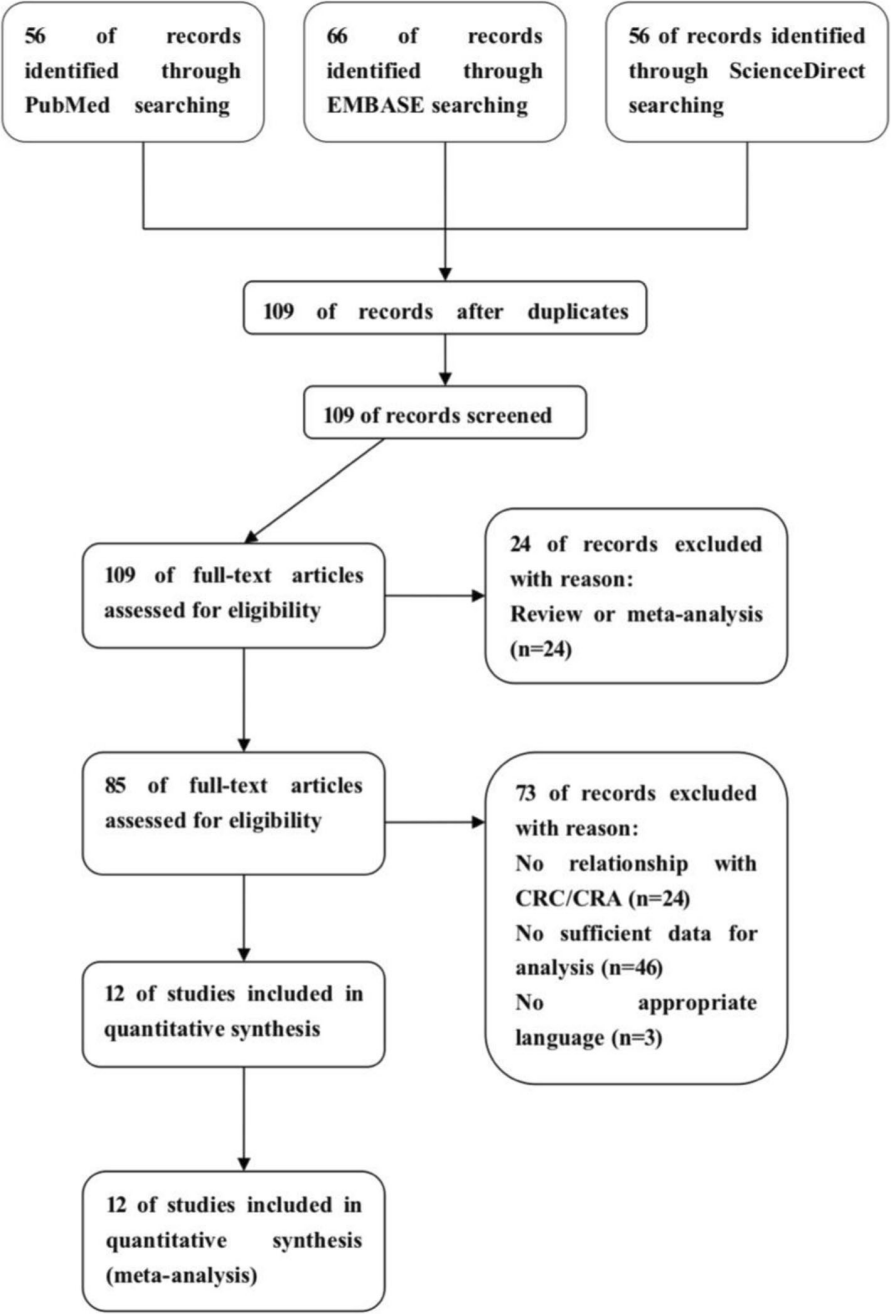
Flow diagram of selection of studies included in the current meta-analysis for the association between rs9929218 polymorphism and CRC/CRA. CRC, colorectal cancer. CRA, colorectal adenomas

Based on our inclusion criteria, we selected eligible articles from the PubMed, Embase, and Science Direct databases. Twelve eligible tests were included in the meta-analysis, involving data from 13 groups, including 6191 patients and 9314 controls associated with CRC and 2001 patients and 2276 controls associated with CRA [14, 15, 19–25]. Genotype distribution was consistent with Hardy-Weinberg equilibrium in all controls studied (*P* > 0.05). The main characteristics of the study are shown in Fig. 1. And the raw data is in Table 1.

**Table 1:** Characteristics of studies included in the meta-analysis

Author	Year	Case			Control			Country	Ethnicity	Source of controls	Type
		AA	AG	GG	AA	AG	GG				
Vonholst	2010	113	700	929	138	648	913	Sweden	European	Mixed	CRC
Rozadilla	2010	65	345	435	83	342	459	Spain	European	Hospital	CRC
Ho	2011	41	261	414	41	261	412	China	Asian	Hospital	CRC
Li	2012	11	57	157	10	83	174	China	Asian	Hospital	CRC
Gira’ldez	2012	10	82	99	108	496	652	Spain	European	Population	CRC
Win	2013	52	144	216	49	183	257	Mixed	Others	Mixed	CRC
Yang	2014	25	214	466	68	566	1168	China	Asian	Population	CRC
Hozyasz	2014	22	102	126	48	222	270	Poland	European	Population	CRC
Burnett	2014	31	183	275	66	321	394	Seattle	Others	Hospital	CRA
Abuli	2016	97	523	706	101	514	651	Spain	European	Hospital	CRA
Ghorbanghli	2016	12	86	88	22	98	109	Holland	European	Population	CRA
Abe	2017	18	188	352	35	325	756	Japan	Asian	Hospital	CRC
Abe	2017	16	145	386	16	172	359	Japan	Asian	Hospital	CRC

*CRC* Colorectal cancer

*CRA* Colorectal adenomas

### Quantitative synthesis

**Fig. 2:**
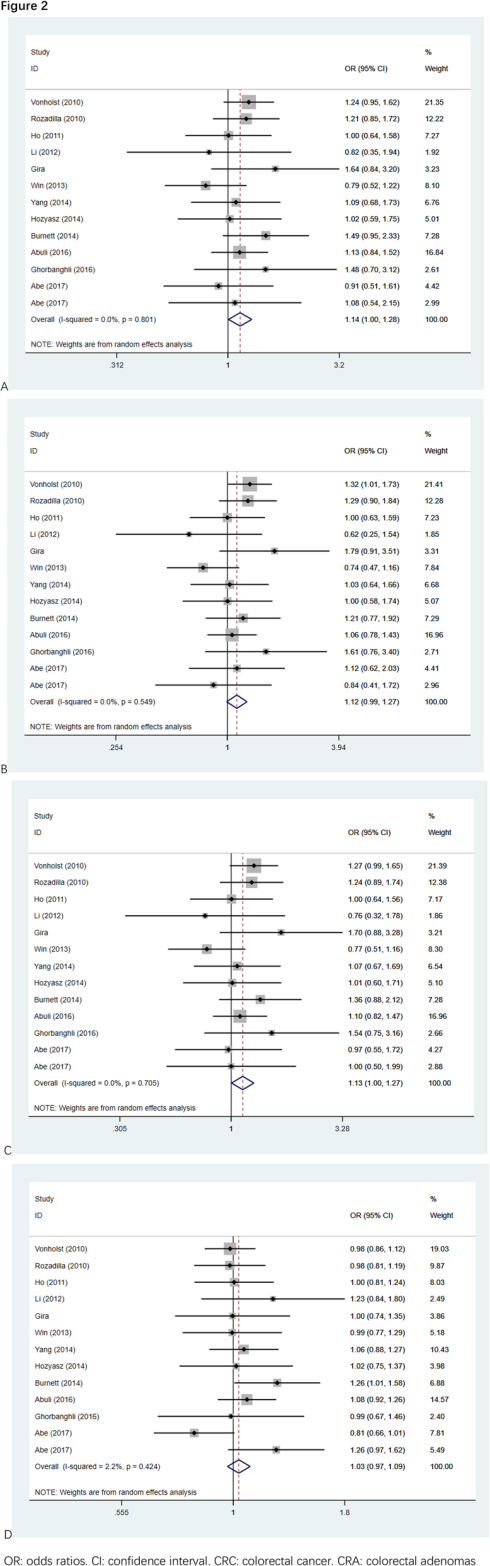
Forest plots for the meta-analysis of rs9929218 polymorphism for overall using a random model. **a** the GG versus AA. **b** the GA versus AA. **c** the dominant model. **d** the recessive model. OR, odds ratios. CI, confidence interval. CRC, colorectal cancer. CRA, colorectal adenomas

We computed the overall OR by the fixed effect model given no significant heterogeneity in all the selected studies. The main results of the meta-analysis for rs9929218 were listed in Table 2. For the increased risk of CRC and rs9929218 polymorphism, there was no significant evidence shows that the correlations were found between them when all eligible studies were integrated into the meta-analysis. As shown in the Fig. 2. For the rs9929218 polymorphism and CRC risk, our meta-analysis showed the general allele to that OR was 1.14 (95%CI 1.00–1.28, *P* = 0.042), and the relevant outcomes underneath the recessive and dominant genetic models were 1.13 (95%CI 1.00–1.27, *P* = 0.044) and 1.03 (95%CI 0.97–1.9, *P* = 0.334). Significant associations were consequently discovered for the dominant model and overall model.

**Table 2:** Stratified analyses of the rs9929218 polymorphism on CRC/CRA risk

Variables	N^a^	Case/Control	GG vs AA			AG vs AA			AG/GG vs AA			GG vs AA/AG		
			OR(95%CI)	*P*^b^	I^2^(%)	OR(96%CI)	*P*^b^	I^2^(%)	OR(97%CI)	*P*^b^	I^2^(%)	OR(98%CI)	*P*^b^	I^2^(%)
Total	13	8192/11590	1.14 (1.00–1.28)^c^	0.042	0	1.12 (0.99–1.27)	0.069	0	1.13 (1.00–1.27)^c^	0.044	0	1.03 (0.97–1.09)	0.334	2.2
Disease type														
CRC	10	6191/9314	1.10 (0.95–1.27)	0.207	0	1.11 (0.95–1.29)	0.201	6.8	1.10 (0.96–1.27)	0.162	0	1.00 (0.94–1.07)	0.909	0
CRA	3	2001/2276	1.25 (0.99–1.58)	0.065	0	1.15 (0.90–1.46)	0.267	0	1.20 (0.96–1.52)	0.113	0	1.12 (0.99–1.26)	0.075	0
Ethnicity														
European	6	4540/5874	1.21 (1.04–1.42)^c^	0.016	0	1.24 (1.06–1.45)^c^	0.008	0	1.22 (1.05–1.42)^c^	0.010	0	1.01 (0.93–1.10)	0.786	0
Asian	5	2751/4446	1.00 (0.78–1.29)	0.996	0	0.97 (0.75–1.26)	0.827	0	0.99 (0.77–1.27)	0.945	0	1.04 (0.89–1.20)	0.642	50.1
Other	2	901/1270	1.08 (0.58–2.00)	0.806	74.3	0.94 (0.58–1.53)	0.818	55.5	1.02 (0.58–1.78)	0.945	70.6	1.13 (0.90–1.43)	0.299	44.6
Source of controls														
hospital	7	4706/5575	1.13 (0.96–1.34)	0.141	0	1.09 (0.92–1.30)	0.318	0	1.12 (0.95–1.32)	0.181	0	1.06 (0.94–1.19)	0.332	47.2
population	4	1332/3827	1.20 (0.90–1.61)	0.213	0	1.21 (0.90–1.63)	0.206	0	1.21 (0.91–1.61)	0.190	0	1.03 (0.90–1.17)	0.653	0
mixed	2	2154/2188	1.03 (0.66–1.59)	0.910	67.3	1.02 (0.58–1.79)	0.951	78.6	1.02 (0.62–1.66)	0.941	75.4	0.99 (0.88–1.11)	0.817	0

a Number of comparisons

b *P* value of Q-test for heterogeneity test

c Random-effects model was used when *P* value for heterogeneity test > 0.05; otherwise, fix-effects model was used

*CRC* Colorectal cancer

*CRA* Colorectal adenomas

**Fig. 3:**
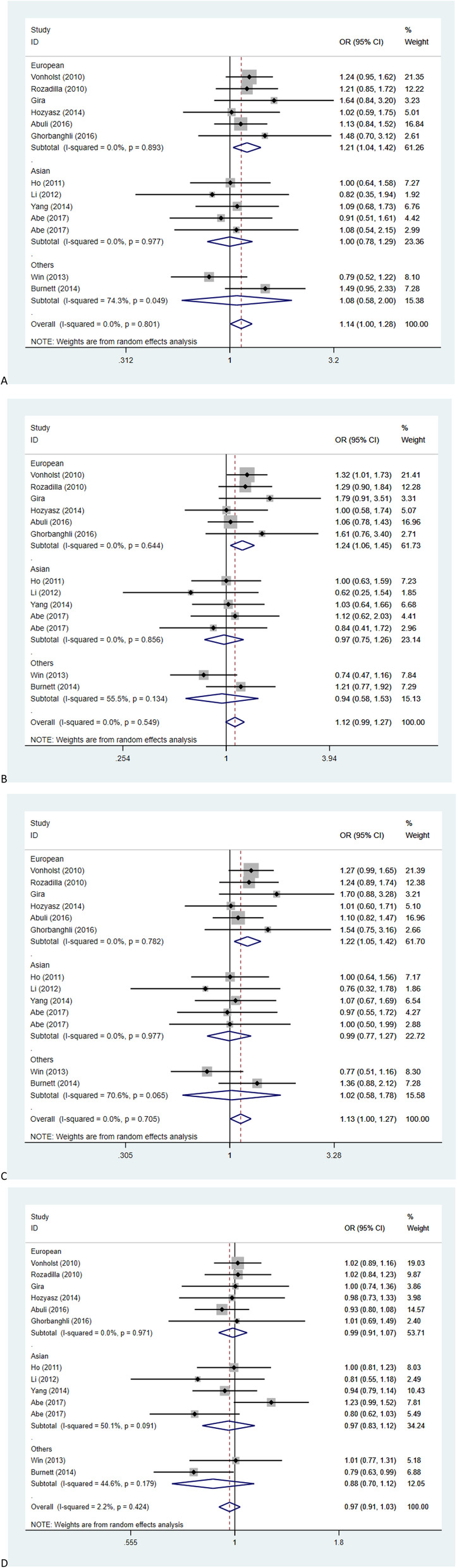
Forest plots for the meta-analysis of rs9929218 polymorphism for ethnicity using a random model. **a** the GG versus AA. **b** the GA versus AA. **c** the dominant model. **d** the recessive model. OR, odds ratios. CI, confidence interval. CRC, colorectal cancer. CRA, colorectal adenomas

A subgroup meta-analysis was further conducted by us in the type of disease, ethnicity, and the source of controls. As shown in the Fig. 3. In stratified by ethnic analysis, a significant association between rs9929218 is supported by the results of the European population and the CRC. In the subgroup analysis, GG genotypes were found to have a significantly higher risk (OR = 1) than AA genotypes in Europeans (OR = 1.21, 95%CI 1.04–1.42, *P* = 0.016), AG versus AA genotype (OR = 1.24, 95%CI 1.06–1.45, *P* = 0.008) and GG/AG versus AA genotype (OR = 1.22, 95%CI 1.05–1.42, *P* = 0.010). Such an association, however, was not obtained in Asian and the Others group. Considering the type of disease and the source of control, the significant association between risk and mutation in patients with CRC / CRA was not found, and there was no such correlation in each group of control groups.

### Test of heterogeneity

According to the test for heterogeneity, there was no statistically significant heterogeneity in the overall comparison (Table 2) and therefore a fixed-effects model was conducted in this meta-analysis. Homozygous comparison (GG vs. AA: P heterogeneity = 0.800, I^2^ = 0%), heterozygote comparison (AG vs. AA: P heterogeneity = 0.548, I^2^ = 0%), the dominant model comparison (GG/AG vs. AA:P heterogeneity = 0.705, I^2^ = 0%) and the recessive model comparison (GG vs. AG/AA:P heterogeneity = 0.424, I^2^ = 0%).

### Sensitivity analyses and publication bias

**Fig. 4:**
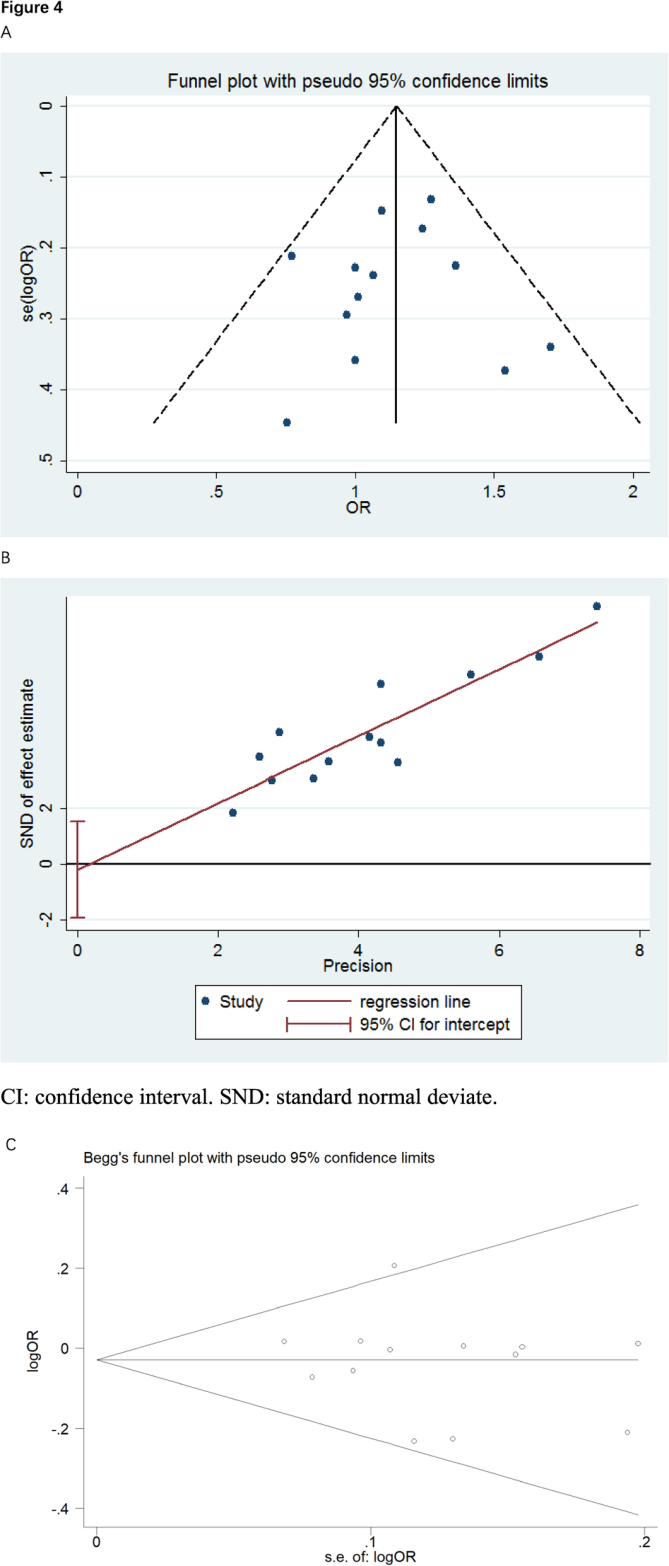
Every point represents a separate study for the indicated association. Funnel plot (**a**) for publication bias analysis of the selected studies investigating the association between rs9929218 polymorphism and CRC/CRA. The X-axis stands for the ORs and the Y-axis is the standard error for each of the 12 studies. A linear regression based approach proposed by Egger test (**b**) and Begg plot (**c**) is used to evaluate the asymmetry of the funnel plot. CI, confidence interval. SND, standard normal deviate

We then evaluated the influence of each study on the combined OR by sequentially omitting each study and the outcomes suggested that none of the studies meaningfully changed the pooled OR, indicating that the pooled OR of this polymorphism was robust. Perform funnel plots and Egger tests to estimate publication bias. As shown in Fig. 4, no significant asymmetry in the shape of the funnel plots was observed, indicating that no significant publication bias was presented in the study.

## Discussion

CRC is one of the gastrointestinal cancers with a high degree of malignancy, aggressiveness, and rapid metastasis. The pathogenic factors of CRC are complicated, as other complex diseases, including both genes and the environment cause [3]. GWAS has different common single nucleotide polymorphism (SNP) associated with colorectal cancer risk. One of SNP with the strong association signal was rs9929218 at 16q22.1 [26, 27]. GWAS and replication studies conducted mostly in European populations but less in other ethnic populations.

Colorectal adenomas (CRA) is well-recognized premalignant lesions of CRC and the majority of CRC are formed from adenomas [3]. It has been reported that many genetic risks of CRC are partially mediated by susceptibility to adenomas. CRC-related SNPs may act by increasing the risk of CRC, CRA, or both [28]. CRA is a recognized precursor of CRC based on epidemiologic, histological, and genetic studies demonstrating shared genetic alterations. Several risk factors are related to the risk of developing CRA that is based on epidemiologic researches including cigarette smoking [29], alcohol intake [30], and obesity [31–34]. Siegert et al. observed significant interactions between alcohol consumption and rs9929218 [35]. In summary, we did a meta-analysis of the relationship between rs9929218 and increased susceptibility to CRC / CRA.

Rs9929218 is situated in the intron zone of CDH1, which encodes a calcium-dependent glycoprotein (E-cadherin). E-cadherin is encoded, a classical calcium adhesion protein, by The CDH1 gene essential for establishing and sustaining cell polarity, tissue constitution, and cell-to-cell adhesion. E-cadherin’s low expression in CRC patients is connected with miserable prognoses, like invasive neoplasm development and metastasis in colorectal cancer [26, 36–38]. It is considered E-cadherin to be an important factor in epithelial-mesenchymal transition (EMT), an important cellular program during tumor cell adhesion, migration, invasion, and metastasis [39]. The EMT is suggested to be the first step in the metastatic of cancer cells by emerging evidence. Loss or reduction of E-cadherin expression is considered to be the primary and most important step in the EMT process, and EMT is a critical step in cancer metastasis [39, 40].

Our meta-analysis involved 12 studies, 13 groups of data, including 8192 cases and 11,590 controls. The combined results demonstrated that the rs9929218 polymorphism was an increased risk factor for CRC in European populations and there was no significant heterogeneity throughout the study. Moreover, sensitivity analysis and publication bias suggest that our results are robust.

When stratified by ethnicity, significant associations between rs9929218 polymorphism and increased incidence of CRC were observed in Europeans. No clear association, however, was observed between Asia and other races in all genetic models, indicating that the conceivable causes are distinctions in habitat and genetic backgrounds. Besides, apart from genetic factors, multiple lifestyles for varying ethnic groups ought to also be reckoned for CRC risk.

Our meta-analysis’s limitations should be regarded in the interpretation of the consequences. Firstly, the number of our case-control study enrolled in our analysis was insufficient. Secondly, we have collected only English literature in this meta-analysis. Thirdly, the involved ethnicities were limited (only Asian, European, and Other population) and further studies of larger sample sizes were needed to explore the influences of different ethnicities. Eventually, this study did not write about some of the potential factors such as age, sex, smoking, alcohol intake, lifestyles, and environmental factors.

## Conclusion

In summary, our meta-analysis studies in different populations confirmed that SNP rs9929218 is significantly associated with CRC risk and that this variant may have a greater impact on Europeans than Asians and Others. Due to the limited research available on the current non-European population, further research is needed, including a broader range of population areas and themes. Furthermore, gene-environment interactions and gene-gene must also be reckoned in succeeding research.

## Data Availability

The datasets generated and analysed during the current study are available in the Embase, PubMed, and ScienceDirect repository, https://pubmed.ncbi.nlm.nih.gov/; https://www.embase.com; https://www.sciencedirect.com.

## Abbreviations

GWAS: Genome-wide association studies
SNPs: Single nucleotide polymorphisms
CRC: Colorectal cancer
CRA: Colorectal adenomas
CDH1: Cadherin 1
ORs: Odds ratios
CIs: Confidence intervals
Cadherin: Calcium-dependent glycoprotein
EMT: Epithelial-mesenchymal transition
PRISMA: Preferred reporting items for systematic review and meta-analysis
